# Perceived community exercise environment and loneliness among middle-aged and older adults in China: psychosocial-behavioral associations, latent profiles, and network analysis

**DOI:** 10.3389/fpubh.2026.1925992

**Published:** 2026-09-21

**Authors:** Junjie Wang, Hao Gou, Xikui Guo, Yanmao Guan

**Affiliations:** 1 School of Physical Education, Ankang University, Ankang, Shaanxi, China; 2 School of Physical Education, Shaanxi Normal University, Xi'an, Shaanxi, China; 3 College of Physical Education, Qiannan Normal University for Nationalities, Duyun, Guizhou, China; 4 Department of Physical Education, Shandong University of Engineering and Vocational Technology, Jinan, Shandong, China

**Keywords:** community exercise environment perception, latent profile analysis, loneliness, middle-aged and older adults, network analysis, physical activity, social support

## Abstract

**Background:**

This study aimed to examine the relationship between perceived community exercise environment and loneliness among middle-aged and older adults in China, investigate the roles of social support and physical activity, and identify distinct environmental perception profiles and key nodes through latent profile analysis and network analysis, thereby providing evidence on factors associated with loneliness in this population.

**Methods:**

A cross-sectional questionnaire survey was conducted from March to April 2026 in community parks, squares, and other public exercise spaces across multiple cities in China. A total of 866 valid responses were included. Participants completed measures of perceived community exercise environment, social support, physical activity, and loneliness. Descriptive statistics, correlation analysis, serial mediation analysis, latent profile analysis, and network analysis were used for data analysis.

**Results:**

Perceived community exercise environment was negatively associated with loneliness and positively associated with social support and physical activity. Social support and physical activity were both negatively associated with loneliness. Serial mediation analysis indicated indirect statistical associations through social support and physical activity, both separately and sequentially. Latent profile analysis identified an environmentally disadvantaged profile and an environmentally advantaged profile. The environmentally disadvantaged profile accounted for 10.51% of the sample and was characterized by lower levels of social support and physical activity and higher loneliness. Network analysis showed that social loneliness and emotional loneliness had the strongest connection. Physical activity, environmental accessibility, and management and maintenance showed relatively strong negative connections with loneliness-related nodes, while social support-related nodes showed relatively high expected influence.

**Conclusion:**

Better perceived community exercise environment was associated with lower loneliness, higher social support, and greater physical activity among middle-aged and older adults in China. The findings suggest that community exercise environments should be understood as multidimensional community resources linked to loneliness through social, behavioral, and environmental associations. Accessible, safe, comfortable, and well-maintained public exercise spaces, together with opportunities for group-based activity and social connection, may be associated with active aging and thereby with psychological wellbeing.

## 1 Introduction

As China's population aging continues to deepen, increasing attention has been paid to the mental health of middle-aged and older adults. According to the National Bureau of Statistics of China, by the end of 2024, the population aged 60 years and above had reached 310.31 million, accounting for 22.0% of the total population, while the population aged 65 years and above had reached 220.23 million, accounting for 15.6% (1). In the context where community-based home care remains the dominant aging model, the quality of community environments, the accessibility of public exercise spaces, and the provision of community services have become important factors affecting healthy living among middle-aged and older adults. In recent years, China has continued to promote the development of “15-min community life circles.” By 2024, 4,335 such life circles had been established, benefiting 107 million people (2). The “15-min community life circle” is a community-based planning concept intended to enable residents to access essential daily services, public facilities, recreational spaces, and health-related resources within approximately a 15-min walk from their homes. For middle-aged and older adults, this concept is particularly relevant because nearby parks, squares, fitness trails, and outdoor exercise facilities can reduce spatial barriers to daily physical activity and provide routine settings for social contact and community participation. Community parks, fitness trails, outdoor fitness equipment, and activity squares constitute important components of this neighborhood service environment and provide nearby settings for physical activity and social interaction among middle-aged and older adults. Meanwhile, a study based on panel data from 30 provinces in China found that urban green space coverage was positively associated with residents' health and may operate through improved air quality and stronger social connections (3).

Loneliness, as a common negative psychological experience among middle-aged and older adults, has been recognized by the World Health Organization as an important public health issue. WHO data indicate that approximately 16% of people worldwide experience loneliness, and about 11.8% of older adults experience loneliness. Social isolation and loneliness can seriously affect physical health, mental health, quality of life, and longevity (4). Previous research has shown that the prevalence of varying degrees of loneliness among older adults in China is 36.6% (5). Therefore, examining the relationship between perceived community exercise environment and loneliness among middle-aged and older adults, as well as its related social and behavioral associations, is of practical significance for understanding how community exercise spaces, social participation, and loneliness are associated.

Although middle-aged adults and older adults represent different life stages, they may share similar neighborhood exercise settings in Chinese communities. In China, the statutory retirement age has traditionally been 60 years for men, 55 years for female cadres, and 50 years for female workers, although a gradual delay in retirement age has recently been introduced. Therefore, adults aged 50–59 years may already be experiencing transition-related changes in work status, family responsibilities, health awareness, and daily activity patterns (6). These changes may increase their reliance on nearby community spaces for exercise, social contact, and leisure activities. For this reason, the present study included both middle-aged adults and older adults, while reporting their age distribution separately. Community parks, squares, and fitness trails therefore provide a common ecological context for examining how environmental perception relates to social support, physical activity, and loneliness across later adulthood (7, 8). The two groups were analyzed within the same framework while their frequencies were reported separately to acknowledge their demographic distinction. However, compared with research on older adults, less is known about whether perceived community exercise environments are already related to loneliness, social support, and physical activity among adults in the transitional stage before or around retirement. By including adults aged 50–59 years together with older adults aged 60 years and above, this study extends the examination of community exercise environments and loneliness to an earlier phase of later adulthood. The conceptual framework of the present study is shown in Figure 1.

**Figure 1:**
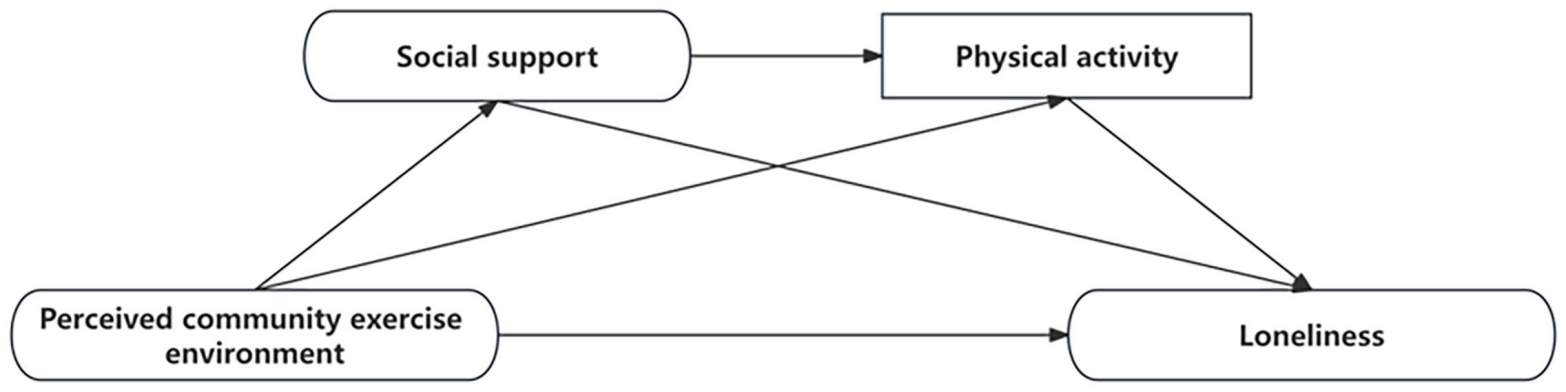
Hypothesized model of the study.

### 1.1 Perceived community exercise environment and loneliness among middle-aged and older adults

The development of loneliness among middle-aged and older adults cannot be attributed solely to aging, changes in family structure, or reduced individual social interaction. According to the social ecological model, health behaviors and mental health outcomes are not determined solely by individual factors, but are shaped by multiple levels of influence, including individual characteristics, social relationships, community environments, and policy contexts (7). For middle-aged and older adults, the community is one of the most important spatial units for daily life, physical activity, and social interaction. Community parks, squares, fitness trails, and other public exercise spaces not only provide places for physical activity, but may also be associated with emotional connection and loneliness because they offer opportunities for outdoor activity, neighborhood contact, and social participation. The World Health Organization's age-friendly cities framework also emphasizes that outdoor spaces and buildings, transportation, social participation, respect and social inclusion, community support, and health services together constitute an important environmental foundation for supporting healthy living in older adults (9).

Neighborhood accessibility and opportunities for community participation are also closely associated with loneliness in later life. In a cross-sectional study of 495 adults aged 60 years and older, participation in community activities statistically mediated the associations of perceived neighborhood accessibility with quality of life and loneliness, indicating that accessible environmental resources may be most beneficial when they facilitate actual participation (10). Complementing this evidence, a four-wave longitudinal study found that loneliness was more consistently associated with older adults' own assessments of neighborhood sociality and conditions than with aggregate assessments reported by other residents (11). From a person–environment fit perspective, these findings suggest that the psychosocial relevance of a neighborhood depends partly on whether its resources correspond to residents' mobility, safety, activity, and social interaction needs.

In China, community accessibility is defined through both spatial proximity and barrier-free usability. The national Standard for Urban Residential Area Planning and Design (GB 50180-2018) organizes residential services into 15-, 10-, and 5-min pedestrian life circles; the 15-min level generally uses a walking service radius of no more than approximately 1 km. In addition, the mandatory General Code for Accessibility of Buildings and Municipal Engineering (GB 55019-2021) requires safe and continuous accessible routes and supporting facilities, while the Law of the People's Republic of China on the Construction of Barrier-Free Environments emphasizes convenient access to roads, buildings, public transport, information, and social services for older adults (12, 13). Thus, accessibility in the present study refers to participants' perceived ease of reaching and using community exercise spaces, rather than an engineering assessment of regulatory compliance.

In recent years, increasing research attention has been given to the relationship between green space, neighborhood environments, loneliness, and mental health among older adults (14). A study based on data from the WHO Study on Global Aging and Adult Health in China found that residential green space was associated with a lower risk of loneliness among middle-aged and older adults in China, and that this relationship was partly mediated by social cohesion (15). Other research has shown that urban green space may influence the mental health of older adults through pathways such as physical activity and social trust (16). These findings suggest that community environments are not merely external background factors in loneliness research, but may represent important ecological factors associated with social participation and mental health among middle-aged and older adults. However, existing studies have mainly focused on general built environments, green spaces, or walking environments. Less attention has been paid to perceived community exercise environments with specific exercise-setting characteristics, and few studies have comprehensively examined their relationship with loneliness from the perspectives of social support, physical activity, and network structure. Therefore, this study proposes the following hypothesis:

### 1.2 The mediating role of social support

The relationship between perceived community exercise environment and loneliness may not result solely from the physical environment, but may also operate through social relational resources. Social support generally refers to the practical assistance, informational resources, emotional care, and sense of belonging that individuals obtain from social relationships, such as family members, friends, neighbors, and social organizations. For middle-aged and older adults, retirement, children leaving home, declines in physical function, and changes in social roles may lead to a shrinking of their original social networks. Without stable social support, they may be more likely to experience social disengagement and loneliness. Previous studies have shown that social isolation and loneliness are closely associated with low social support, fewer social connections, and poorer mental health (17). A systematic review of interventions for loneliness among older adults reported that interventions combining social support, opportunities for social interaction, and social-skills development tended to be more effective than approaches addressing only one component. A particular advantage of expanding social opportunities is that regular group activities and repeated face-to-face contact enable older adults to meet others, develop relationships, and convert social contact into sustained social support (18).

Community exercise environments may provide important settings for the formation of social support. A community exercise space perceived as accessible, safe, comfortable, and well maintained can not only increase the frequency of outdoor activities among middle-aged and older adults, but also create opportunities for interaction with neighbors, peers, and other community members. In public spaces such as community parks, squares, and fitness trails, middle-aged and older adults may obtain support through exercising together, exchanging health information, participating in group activities, and building stable peer relationships. Previous studies have shown that neighborhood social environments, perceived community safety, neighborhood connections, and social trust are closely related to the mental health of older adults. Better neighborhood safety and social trust have been associated with fewer negative emotions and better mental health. (19). In addition, green spaces may also be associated with physical and mental health through their links with social contact, social cohesion, and physical activity. (20, 21).

Collectively, previous studies indicate that accessible, safe, and socially supportive neighborhood environments are associated with more frequent social interaction and stronger social support, while greater social support is associated with lower loneliness among older adults (17, 19–21).

### 1.3 The mediating role of physical activity

Previous studies have found a systematic negative association between loneliness and physical activity among older adults. In particular, green space and neighborhood environments may contribute to mental health through social cohesion, physical activity, and reduced loneliness (22, 23). This relationship may partly reflect the social opportunities embedded in group- or community-based activity, because companionship and functional social support can facilitate participation and strengthen social connection (24–26). Systematic reviews have identified recreational facilities, parks and public open spaces, street connectivity, safety, walkability, and access to destinations as correlates of physical activity among older adults, although individual factors such as age and health status also remain relevant (8, 27, 28). Although associations between the built environment and physical activity, and between physical activity and loneliness, have each been examined, evidence integrating perceived community exercise environment, physical activity, and loneliness within a single analytical framework remains limited, particularly in Chinese community samples. The present study therefore examines whether physical activity statistically accounts for part of the association between perceived community exercise environment and loneliness, without assuming a causal pathway.

### 1.4 The serial mediating role of social support and physical activity

The relationship between perceived community exercise environment and loneliness among middle-aged and older adults may not operate through a single pathway, but rather through a sequential process involving social relational resources and health behavior participation. The social ecological model emphasizes that health behaviors and mental health outcomes are jointly shaped by environmental conditions, social relationships, and individual behaviors (7, 29). In the context of community life among middle-aged and older adults, a community exercise environment perceived as accessible, safe, comfortable, and well maintained may be associated with more opportunities for neighborhood contact and group activities, making it easier for individuals to obtain emotional companionship, information exchange, and practical assistance. These experiences may be linked to higher levels of social support, which may also be associated with motivation, confidence, and persistence in physical activity (30).

Social support is closely related to physical activity among older adults. Encouragement, companionship, reminders, and practical assistance from family members, friends, neighbors, and peers are often associated with fewer perceived exercise barriers and better exercise adherence. Previous reviews have shown that social support is positively associated with physical activity levels among older adults, and that support from family members, friends, and peers is positively associated with participation in physical activity. In community exercise settings, exercising with others, joining group activities, and receiving peer support are often associated with higher physical activity participation, but also make physical activity a socially embedded behavior. Therefore, middle-aged and older adults with more positive perceptions of community exercise environments may first obtain greater social support through community interaction and then participate more actively in physical activity with the support of these social resources (26).

Physical activity may further serve as an important behavioral pathway through which social support is associated with loneliness. In the serial pathway proposed in this study, social support is expected to facilitate physical activity participation, and physical activity may then be associated with lower loneliness through improved function, emotional state, and social participation. Community-based exercise in shared public spaces is a common form of later-life physical activity in China, rather than merely a substitute for individualized exercise in gyms or sports centers. Observational and qualitative studies show that older adults regularly use neighborhood parks, residential squares, and outdoor fitness areas for walking, Tai Chi, square dancing, and apparatus-based exercise. These activities are often low-cost, informal, repeated, and group-based, allowing public exercise spaces to function simultaneously as activity settings and sites for social networks, mutual support, and belonging (31). This culturally embedded pattern makes the accessibility, safety, comfort, and maintenance of community exercise spaces particularly relevant to the everyday activity and social participation of middle-aged and older adults in China.

### 1.5 Latent profile and network analysis

Most studies of community environments, physical activity, and loneliness have adopted variable-centered approaches that estimate overall associations across the whole sample. However, middle-aged and older adults may differ in their perceived accessibility, facility convenience, safety, comfort, natural environment, and management and maintenance. Latent profile analysis can identify such subgroups and clarify whether environmentally disadvantaged profiles show lower social support, lower physical activity, and higher loneliness. Latent profile analysis is a person-centered method that classifies individuals according to similar response patterns and supports interpretation of profile differences rather than only average effects. Model selection was based on fit indices, classification accuracy, class size, and theoretical interpretability (32–34).

Because latent profile analysis does not reveal fine-grained links among specific dimensions, network analysis was also used. Network analysis treats environmental, social, behavioral, and loneliness variables as connected nodes and can identify direct conditional associations and potentially important nodes within the system (35–37). Together, these approaches allow the present study to examine perceived community exercise environment and loneliness from complementary perspectives: overall sample-level associations, social-behavioral pathways, subgroup heterogeneity, and dimension-level network connections.

## 2 Materials and methods

### 2.1 Study hypotheses

Based on the literature review and the overall study rationale, the following hypotheses were tested:

H1: Perceived community exercise environment is negatively associated with loneliness among middle-aged and older adults.

H2: Social support mediates the relationship between perceived community exercise environment and loneliness among middle-aged and older adults.

H3: Physical activity mediates the relationship between perceived community exercise environment and loneliness among middle-aged and older adults.

H4: Social support and physical activity play a serial mediating role in the relationship between perceived community exercise environment and loneliness among middle-aged and older adults.

### 2.2 Participants

This study adopted a cross-sectional questionnaire survey design and was conducted from March to April 2026 in community parks, squares, fitness trails, and other public exercise spaces in China. The survey covered six cities, including Xi'an and Ankang in Shaanxi Province, Shenyang in Liaoning Province, Chongqing, Taiyuan in Shanxi Province, and Yinchuan in Ningxia Hui Autonomous Region. The final valid sample was distributed across the six cities as follows: Xi'an (*n* = 150), Ankang (*n* = 146), Shenyang (*n* = 142), Chongqing (*n* = 144), Taiyuan (*n* = 140), and Yinchuan (*n* = 144). Recruitment procedures were generally consistent across study sites. In each city, trained investigators recruited eligible middle-aged and older adults on site in community parks, squares, fitness trails, and other public exercise spaces, explained the study purpose and informed consent procedures, and administered the questionnaire face to face. The target population consisted mainly of middle-aged and older adults in China who had experience participating in community-based exercise.

The questionnaire included demographic information, perceived community exercise environment, social support, physical activity, and loneliness. The inclusion criteria were as follows: (1) aged 50 years or above; (2) having participated in exercise in community parks, squares, fitness trails, or other public exercise spaces within the past 3 months; (3) being able to understand the questionnaire and complete it; and (4) voluntarily participating in the study and providing informed consent. The exclusion criteria were as follows: (1) questionnaire completion rate below 80%; (2) obvious patterned responses or highly identical answers; (3) abnormally short response time; (4) serious missing or invalid responses for core variables; and (5) inability to complete the questionnaire due to severe cognitive, hearing, or visual impairment.

A total of 893 questionnaires were collected. After excluding 27 invalid questionnaires according to the predefined quality control criteria, 866 valid questionnaires were included in the final analysis, yielding an effective response rate of 96.98%. In the final sample, 432 participants were male, accounting for 49.9%, and 434 were female, accounting for 50.1%. Participants ranged in age from 50 to 89 years (M = 69.70 years). Based on the commonly used age threshold in the Chinese context, 204 participants were middle-aged adults aged 50–59 years (23.56%), and 662 were older adults aged 60 years or above (76.44%). Both groups were included because they used the same types of community exercise spaces and were examined within a shared later-adulthood ecological framework; their frequencies are reported separately to preserve the distinction between the two populations. To further describe the age distribution, the sample was dominated by older adults aged 60 years or above, while participants aged 50–59 years represented a smaller but meaningful middle-aged subgroup within the transition to later adulthood.

### 2.3 Procedure

Before the formal survey, the research team provided standardized training for the investigators. The training covered the explanation of the research purpose, questionnaire distribution procedures, informed consent procedures, principles for explaining questionnaire items, data confidentiality requirements, identification of invalid questionnaires, and handling of unexpected situations during fieldwork, in order to ensure consistency in the survey process and data quality. During the survey, researchers first explained the purpose of the study, the principles of anonymity and voluntary participation, the use of data solely for scientific research, and participants' right to withdraw from the study at any time. After informed consent was obtained, participants completed the questionnaire independently. For middle-aged and older adults with limited reading ability or difficulty completing the questionnaire, trained investigators read the items aloud in a neutral tone and assisted them in completing the questionnaire without guiding their responses.

### 2.4 Measurements

#### 2.4.1 Development and assessment of the perceived community exercise environment questionnaire

This study used a perceived community exercise environment scale developed for this study to measure middle-aged and older adults' subjective evaluation of the community exercise environment. Perceived community exercise environment refers to individuals' overall evaluation of accessibility, facility convenience, safety, comfort, natural environment, and management and maintenance in community parks, squares, fitness trails, and other public exercise spaces. The development of this scale was mainly based on the following considerations. First, the social ecological model and research on active living environments suggest that individual physical activity and health behaviors are influenced not only by personal factors, but also by multiple levels of social, built, and policy environments (38, 39). Second, the Neighborhood Environment Walkability Scale and its abbreviated versions provide important references for measuring dimensions such as accessibility, facility convenience, safety, walking environment, and community open spaces (40, 41). Third, the World Health Organization's age-friendly cities framework emphasizes that outdoor spaces, accessibility of public facilities, safety, social participation, and community support are important environmental foundations for promoting healthy living among older adults (42). Considering that physical activity among middle-aged and older adults in China is strongly embedded in community settings, dependent on specific exercise spaces, and closely related to everyday life, this study developed a perceived community exercise environment scale suitable for middle-aged and older community exercisers in China through qualitative interviews, expert consultation, and a pilot test, based on the above theoretical and measurement foundations.

To make the measurement content clearer, the six dimensions were defined as follows. Accessibility assessed whether community exercise spaces were easy to reach and enter. Facility convenience assessed the availability, usability, and convenience of exercise facilities. Safety assessed perceived risks related to lighting, ground surfaces, anti-slip conditions, and the surrounding exercise environment. Comfort assessed cleanliness, crowding, noise, and the overall ease of exercising in the space. Natural environment assessed greenery, shade, air quality, and landscape features. Management and maintenance assessed regular inspection and repair of facilities, cleanliness and sanitation of the exercise site, order management, and timely handling of damaged equipment or environmental problems. Each dimension was represented by three items.

During the item generation stage, the research team first systematically reviewed the literature on perceived neighborhood environment, physical activity environments, age-friendly communities, community sports participation, and outdoor activity spaces for older adults. Based on the practical characteristics of community exercise among middle-aged and older adults, a preliminary theoretical framework of perceived community exercise environment was constructed. This framework included six dimensions: accessibility, facility convenience, safety, comfort, natural environment, and management and maintenance. Subsequently, semi-structured interviews were conducted to collect middle-aged and older adults' real experiences of community exercise spaces. The interviewees included middle-aged and older adults who had long participated in exercise in community parks, squares, fitness trails, and other public exercise spaces, as well as individuals familiar with community sports, older adult sports, and public exercise space services. The interviews focused mainly on exercise space selection, facility use, environmental safety, exercise comfort, natural landscapes, peer interaction, venue management and maintenance, and barriers to sustained participation. Following the procedures of grounded theory, including open coding, axial coding, and selective coding, the research team organized and summarized the interview data and developed an initial item pool (43). The qualitative interviews were conducted before the formal survey and were used only for item generation and wording refinement. Interview information was not included in the final quantitative analysis.

To improve the content validity of the scale, this study used the Delphi expert consultation method to revise and screen the initial items (44). Experts from fields including sports science, older adult sports, community sports, public health, environmental psychology, and psychometrics were invited to participate in two rounds of consultation. The experts mainly evaluated the rationality of the dimensional structure, representativeness of item content, clarity of item wording, applicability to middle-aged and older adults, and the presence of repetition or ambiguity. Based on expert feedback, the research team deleted items with overlapping content or unclear theoretical orientation, revised items that were too abstract or difficult for middle-aged and older adults to understand, and added items that better reflected real community exercise contexts. After two rounds of expert consultation and discussion within the research team, the perceived community exercise environment scale for the pilot test was finalized. The Delphi consultation was conducted independently from the formal participant survey; experts were invited to evaluate the dimensional structure and item content, and they did not participate as survey respondents.

The pilot version of the scale included 18 items across six dimensions, with three items for each dimension. A 5-point Likert scale was used, ranging from 1 = “strongly disagree” to 5 = “strongly agree.” Higher scores indicated more positive perceptions across accessibility, facility convenience, safety, comfort, natural environment, and management and maintenance. In the main statistical analyses, the mean score of the 18 items was used to represent the overall level of perceived community exercise environment. In the latent profile analysis and network analysis, the mean scores of the six dimensions were further used as analytical indicators. The complete 18 items and scoring instructions are provided in Supplementary Table S1.

A pilot test was conducted among 240 middle-aged and older community exercisers. The item analysis showed that the absolute critical ratio values of the 18 items ranged from 6.876 to 11.432, all exceeding 3.0. The item-total correlation coefficients ranged from 0.472 to 0.587, all above 0.40, indicating good item discrimination. Reliability analysis showed that the overall Cronbach's α coefficient of the perceived community exercise environment scale in the pilot sample was 0.841, and the Cronbach's α coefficients for the six dimensions ranged from 0.916 to 0.939, indicating good internal consistency (45). The pilot sample was independent of the formal survey sample and was not included in the final analysis of 866 valid questionnaires.

Exploratory factor analysis showed that the Kaiser–Meyer–Olkin value was 0.757, and Bartlett's test of sphericity was significant, χ^2^ = 3535.921, df = 153, *p* < 0.001, indicating that the data were suitable for factor analysis. Principal component analysis with varimax rotation extracted six factors with eigenvalues greater than 1, explaining 87.18% of the cumulative variance. Factor loadings ranged from 0.895 to 0.938, and no obvious cross-loadings were observed. The extracted factor structure was consistent with the theoretical expectation (46). Confirmatory factor analysis further supported the six-factor structure of the scale. The model fit indices were as follows: χ^2^/df = 1.893, RMSEA = 0.061, SRMR = 0.053, CFI = 0.969, and TLI = 0.961, indicating good model fit.

In the formal survey, 866 valid questionnaires were included. In the formal sample, the overall Cronbach's α coefficient of the perceived community exercise environment scale was 0.852. The Cronbach's α coefficients for the six dimensions were 0.878 for accessibility, 0.867 for facility convenience, 0.875 for safety, 0.861 for comfort, 0.912 for natural environment, and 0.902 for management and maintenance. These results indicate that the scale maintained good internal consistency in the formal sample and can be used to evaluate perceived community exercise environment among middle-aged and older adults in China.

#### 2.4.2 Social support

Social support was measured using the Social Support Rating Scale for Older Adults (47). This scale is applicable to older adults in China and consists of 14 items across four dimensions: instrumental support, informational support, perceived support, and support utilization. It mainly assesses older adults' practical assistance, access to information, emotional care, and active use of support resources. The scale uses a 5-point Likert scoring method, ranging from 1 = “never” to 5 = “always.” All 14 items are positively scored, with higher scores indicating higher levels of social support. The formal version of the scale has a Cronbach's α coefficient of 0.848 and a split-half reliability of 0.899. In the present study, the overall Cronbach's α coefficient of the scale was 0.848, indicating good internal consistency. Although the scale was originally developed for older adults, its content focuses on general social support resources, including instrumental support, informational support, perceived support, and support utilization. These domains reflect common social needs in adulthood, especially among adults aged 50–59 years who are approaching retirement and increasingly participate in community-based activities. In addition, the scale showed good internal consistency in the full sample of the present study, supporting its use in this mixed middle-aged and older adult sample.

#### 2.4.3 Physical activity

Physical activity was measured using the Physical Activity Rating Scale-3 (PARS-3) (48). The scale consists of three items: exercise intensity, exercise duration, and exercise frequency, and is used to assess individuals' recent level of physical activity participation. In this study, exercise intensity, exercise duration, and exercise frequency were all scored on a 1–5 scale. The mean score of the three items was used to represent the level of physical activity participation. Because PARS-3 assesses general exercise intensity, duration, and frequency, it is applicable to both middle-aged and older adults in community exercise contexts. Although PARS-3 is commonly scored by multiplying exercise intensity, duration, and frequency, the mean score was used in the main analyses because all three items were coded on the same 1–5 response scale and the mean score provided an interpretable metric consistent with the other scale-based variables in the mediation, latent profile, and network analyses.

The PARS-3 is commonly used to calculate physical activity volume using the formula “exercise intensity × exercise duration × exercise frequency.” However, to facilitate comparison with the mean scores of other psychological scales, this study used the average score of the three items—exercise intensity, exercise duration, and exercise frequency—as the main indicator of physical activity participation in the statistical analyses. Higher scores indicated higher levels of physical activity participation. In the present study, the Cronbach's α coefficient of the PARS-3 was 0.941, indicating acceptable internal consistency.

#### 2.4.4 Loneliness

Loneliness was measured using the Loneliness Scale for Older Adults (47). This scale was developed for older adults in China and consists of 10 items across two dimensions: social loneliness and emotional loneliness. It mainly assesses older adults' loneliness experiences related to insufficient social interaction, lack of social connections, and inadequate emotional companionship. The scale uses a 5-point Likert scoring method, ranging from 1 = “never” to 5 = “always,” with higher scores indicating higher levels of loneliness. The formal version of the scale has a Cronbach's α coefficient of 0.891 and a split-half reliability of 0.889. In the present study, the overall Cronbach's α coefficient of the scale was 0.898, indicating good internal consistency. Although this scale was developed for older adults, the items assess general experiences of social and emotional loneliness rather than age-specific symptoms only. Therefore, the scale was considered applicable to participants aged 50–59 years in this study, particularly because these participants shared the same community exercise contexts as the older adult group. The good internal consistency observed in the present sample further supported its use.

### 2.5 Statistical analysis

SPSS 26.0 was used for descriptive statistics, reliability analysis, common method bias testing, Pearson correlation analysis, and serial mediation analysis. The PROCESS macro was used to conduct serial mediation analysis, with Model 6 specified, and 5,000 bootstrap samples were used to calculate the 95% confidence intervals for indirect effects (49). Mplus 8.3 was used for latent profile analysis. R software was used for network analysis, mainly using the R packages qgraph and bootnet to perform network estimation, centrality analysis, and stability testing [R Studio (50); R Core (51)]. All statistical tests were two-tailed, and the significance level was set at p < 0.05.

#### 2.5.1 Preliminary analysis

Descriptive statistics were used to summarize the sample characteristics and main variables. Because all included scale items and dimensions were coded on a 1–5 response scale and all valid respondents completed the relevant items, item mean scores placed the variables on a comparable metric. Therefore, the item mean scores of each scale or dimension were used as analytical indicators in the main statistical analyses. Cronbach's **α** coefficients were used to evaluate the internal consistency of each scale and dimension. Pearson correlation analysis was conducted to examine the bivariate relationships among perceived community exercise environment, social support, physical activity, and loneliness. As the data in this study were mainly collected through self-report questionnaires, Harman's single-factor test was used to preliminarily assess common method bias (52).

#### 2.5.2 Mediation analysis

To examine the mediating roles of social support and physical activity in the relationship between perceived community exercise environment and loneliness, a serial mediation model was constructed. Perceived community exercise environment was treated as the independent variable, loneliness as the dependent variable, and social support and physical activity as mediating variables. Social support was specified as the first mediator, and physical activity was specified as the second mediator.

The mediating effects were tested using the bias-corrected bootstrap method with 5,000 repeated samples, and 95% confidence intervals were calculated for the indirect effects. An indirect effect was considered statistically significant when the 95% confidence interval did not include zero. Three indirect pathways were examined: (1) perceived community exercise environment → social support → loneliness; (2) perceived community exercise environment → physical activity → loneliness; and (3) perceived community exercise environment → social support → physical activity → loneliness. Because this study used a cross-sectional design, the mediation analysis was used only to describe statistical indirect associations among variables and did not support strict causal inference.

#### 2.5.3 Latent profile analysis

To identify potential heterogeneity in perceived community exercise environment among middle-aged and older adults, latent profile analysis was conducted using the six dimensions of the perceived community exercise environment scale as profile indicators, including accessibility, facility convenience, safety, comfort, natural environment, and management and maintenance. Before analysis, the scores of the six dimensions were standardized, and one- to five-profile models were fitted sequentially.

Model selection was mainly based on AIC, BIC, aBIC, Entropy, LMR, and BLRT, as well as class proportions and theoretical interpretability (53). In general, lower AIC, BIC, and aBIC values indicate better model fit; Entropy values closer to 1 indicate higher classification accuracy; and significant LMR and BLRT results suggest that a k-profile model fits the data better than a k−1 profile model. To avoid over-extraction of profiles, class proportions and practical interpretability were also considered in determining the optimal model. After the optimal latent profile model was identified, differences in social support, physical activity, and loneliness were further compared across the different profile groups.

#### 2.5.4 Network analysis

To further reveal fine-grained associations among the dimensions of perceived community exercise environment, social support, physical activity, and loneliness, a dimension-level network was constructed. The network nodes included the six dimensions of perceived community exercise environment, namely accessibility, facility convenience, safety, comfort, natural environment, and management and maintenance; the four dimensions of social support, namely instrumental support, informational support, perceived support, and support utilization; physical activity score; and the two dimensions of loneliness, namely social loneliness and emotional loneliness.

As these nodes were mainly continuous variables or dimensional mean scores, a regularized partial correlation network was used to estimate the relationships among nodes. Edges in the network represented conditional associations between two nodes after controlling for all other nodes. The EBICglasso method was used to estimate the network structure, with the tuning parameter γ set to 0.50, and the Fruchterman–Reingold algorithm was used for network visualization. Considering that the network contained both positive and negative connections, expected influence was used as the main centrality indicator to identify key nodes in the network (37). Network robustness was evaluated using bootstrap methods, including nonparametric bootstrap edge-weight difference testing and case-dropping bootstrap centrality stability testing.

## 3 Results

### 3.1 Sample characteristics

Among the 866 valid participants, 432 were men (49.88%) and 434 were women (50.12%). Based on the age grouping reported above, 204 participants were middle-aged adults aged 50–59 years (23.56%), and 662 were older adults aged 60 years or above (76.44%). Regarding marital status, 614 participants (70.90%) were married, and 252 (29.10%) were unmarried, divorced, widowed, or otherwise not currently married.

### 3.2 Common method bias test

The data in this study were mainly collected through self-report questionnaires completed by middle-aged and older adults, which may be subject to common method bias. To examine whether common method bias had a serious influence on the study results, Harman's single-factor test was used for preliminary assessment (54). Items related to perceived community exercise environment, social support, physical activity, and loneliness were entered into an unrotated exploratory factor analysis. The results showed that 13 factors had eigenvalues greater than 1, and the first factor explained 24.62% of the variance, which was below the critical threshold of 40%. These results suggest that no serious common method bias was detected in this study.

### 3.3 Correlation analysis among variables

Descriptive statistics and Pearson correlation analysis were conducted for perceived community exercise environment, social support, physical activity, and loneliness, and the results are shown in Table 1. Perceived community exercise environment was significantly positively correlated with social support and physical activity, and significantly negatively correlated with loneliness. Social support was significantly positively correlated with physical activity, and both social support and physical activity were significantly negatively correlated with loneliness. The directions of the correlations among variables were consistent with theoretical expectations, providing a statistical basis for the subsequent serial mediation model.

**Table 1:** Descriptive statistics and correlation analysis of the main variables.

Variable	M	SD	1	2	3	4
CSEP	3.05	0.75	1			
SS	3.01	0.82	0.556^***^	1		
PA	3.04	1.32	0.329^***^	0.345^***^	1	
Loneliness	3.01	1.04	−0.572^***^	−0.483^***^	−0.402^***^	1

N = 866; M, mean; SD, standard deviation; CSEP, perceived community exercise environment; SS, social support; PA, physical activity; ^***^ p < 0.001.

### 3.4 Mediation analysis

To further examine the statistical mediating roles of social support and physical activity in the relationship between perceived community exercise environment and loneliness, this study constructed a serial mediation model based on the model specified in the Statistical analysis section (55). Perceived community exercise environment was treated as the independent variable, loneliness as the outcome variable, and social support and physical activity as mediating variables, with social support specified as the first mediator and physical activity as the second mediator. Bootstrap resampling was performed 5,000 times to calculate the 95% confidence intervals for the indirect effects.

The regression results of the serial mediation model are shown in Table 2. Perceived community exercise environment was significantly positively associated with social support. After perceived community exercise environment and social support were entered simultaneously, perceived community exercise environment was significantly positively associated with physical activity, and social support was also significantly positively associated with physical activity. In the model with loneliness as the outcome variable, perceived community exercise environment remained significantly negatively associated with loneliness, while social support and physical activity were both significantly negatively associated with loneliness.

**Table 2:** Regression results of the serial mediation model.

Dependent variable	Predictor	R	R^2^	F	B	SE	t	95% CI	*p*
SS	CSEP	0.556	0.309	387.097	0.609	0.031	19.675	[0.548, 0.669]	< 0.001
PA	CSEP	0.382	0.146	73.716	0.350	0.067	5.233	[0.218, 0.481]	< 0.001
PA	SS	0.382	0.146	73.716	0.378	0.061	6.195	[0.258, 0.498]	< 0.001
Loneliness	CSEP	0.635	0.403	193.666	−0.556	0.045	−12.407	[-0.644,−0.468]	< 0.001
Loneliness	SS	0.635	0.403	193.666	−0.242	0.041	−5.864	[-0.323,−0.161]	< 0.001
Loneliness	PA	0.635	0.403	193.666	−0.162	0.022	−7.210	[-0.206,−0.118]	< 0.001

B, unstandardized regression coefficient; CI, confidence interval; CSEP, perceived community exercise environment; SS, social support; PA, physical activity.

The bootstrap mediation effect test results are shown in Table 3. The total indirect effect of social support and physical activity in the relationship between perceived community exercise environment and loneliness was significant. Further decomposition of the indirect pathways showed that the 95% confidence intervals for the three indirect pathways did not include zero: perceived community exercise environment -> social support -> loneliness; perceived community exercise environment -> physical activity -> loneliness; and perceived community exercise environment -> social support -> physical activity -> loneliness. These results indicate that the separate indirect effects of social support and physical activity and their serial indirect effect were statistically significant. Sensitivity analysis using the conventional PARS-3 product score showed the same substantive pattern as the main analysis. The product score was highly correlated with the mean score of the three PARS-3 items and showed the same directions of association with perceived community exercise environment, social support, and loneliness. These results are provided in Supplementary Table S8.

**Table 3:** Mediation effects of social support and physical activity.

Pathway	Effect	BootSE	BootLLCI	BootULCI
Total indirect effect	−0.241	0.031	−0.302	−0.184
CSEP -> SS -> loneliness	−0.147	0.029	−0.205	−0.091
CSEP -> PA -> loneliness	−0.057	0.013	−0.083	−0.033
CSEP -> SS -> PA -> loneliness	−0.037	0.008	−0.056	−0.022

CSEP, perceived community exercise environment; SS, social support; PA, physical activity. Bootstrap resampling was performed 5,000 times. BootLLCI and BootULCI represent the lower and upper bounds of the bootstrap 95% confidence interval, respectively. A confidence interval that does not include zero indicates a significant indirect effect.

### 3.5 Latent profile analysis

In terms of model fit indices, AIC, BIC, and aBIC continued to decrease as the number of latent profiles increased, and both the LMR and BLRT tests were significant, indicating that adding profiles improved model fit. However, the determination of the latent profile model should not rely only on statistical fit indices, but should also consider classification accuracy, class proportions, model parsimony, and theoretical interpretability. Compared with the three-, four-, and five-profile models, the two-profile model had the highest Entropy value (0.977), indicating the best classification accuracy. At the same time, after additional profiles were added, the newly identified classes mainly represented further subdivisions of the high environmental perception group, and the trends across classes were largely consistent, without forming new profiles with clear theoretical distinctions. Thus, the additional explanatory value was limited. Considering statistical indices, classification quality, and theoretical interpretability, this study selected the two-profile model as the optimal latent profile model (56, 57). It should be noted that the latent profile model was established only on the basis of the six dimensions of perceived community exercise environment, while social support, physical activity, and loneliness were used only to describe the external characteristics of different profiles. The fit indices for the latent profile models are shown in Table 4.

**Table 4:** Comparison of fit indices for latent profile models.

Class	AIC	BIC	aBIC	Entropy	LMR p	BLRT p	Class Probability (%)
1	37,145.291	37,269.152	37,186.582	/	/	/	100.000
2	35,758.164	35,948.719	35,821.689	0.977	< 0.001	< 0.001	10.508/89.492
3	35,076.742	35,333.992	35,162.502	0.938	< 0.001	< 0.001	45.497/10.162/44.342
4	34,519.052	34,842.996	34,627.046	0.955	< 0.001	< 0.001	10.277/43.303/29.446/16.975
5	34,405.914	34,796.552	34,536.141	0.932	< 0.001	< 0.001	10.277/13.626/30.023/29.330/16.744

Figure 2 presents the mean score patterns of the two latent profiles across the study variables. Class 1 scored substantially lower than Class 2 on all six dimensions of perceived community exercise environment. It also showed lower levels of social support and physical activity, but higher scores for social loneliness and emotional loneliness. This pattern indicates that this group was generally in a relatively disadvantaged position in terms of perceived community exercise resources and related health resources; therefore, it was named the Environmentally Disadvantaged Profile (EDP). The EDP included 91 participants, accounting for 10.51% of the sample. Class 2 showed higher scores across all dimensions of perceived community exercise environment, accompanied by higher levels of social support and physical activity and lower loneliness. This group was therefore named the Environmentally Advantaged Profile (EAP), reflecting its overall advantage in perceived community exercise environment. The EAP included 775 participants, accounting for 89.49% of the sample.

**Figure 2:**
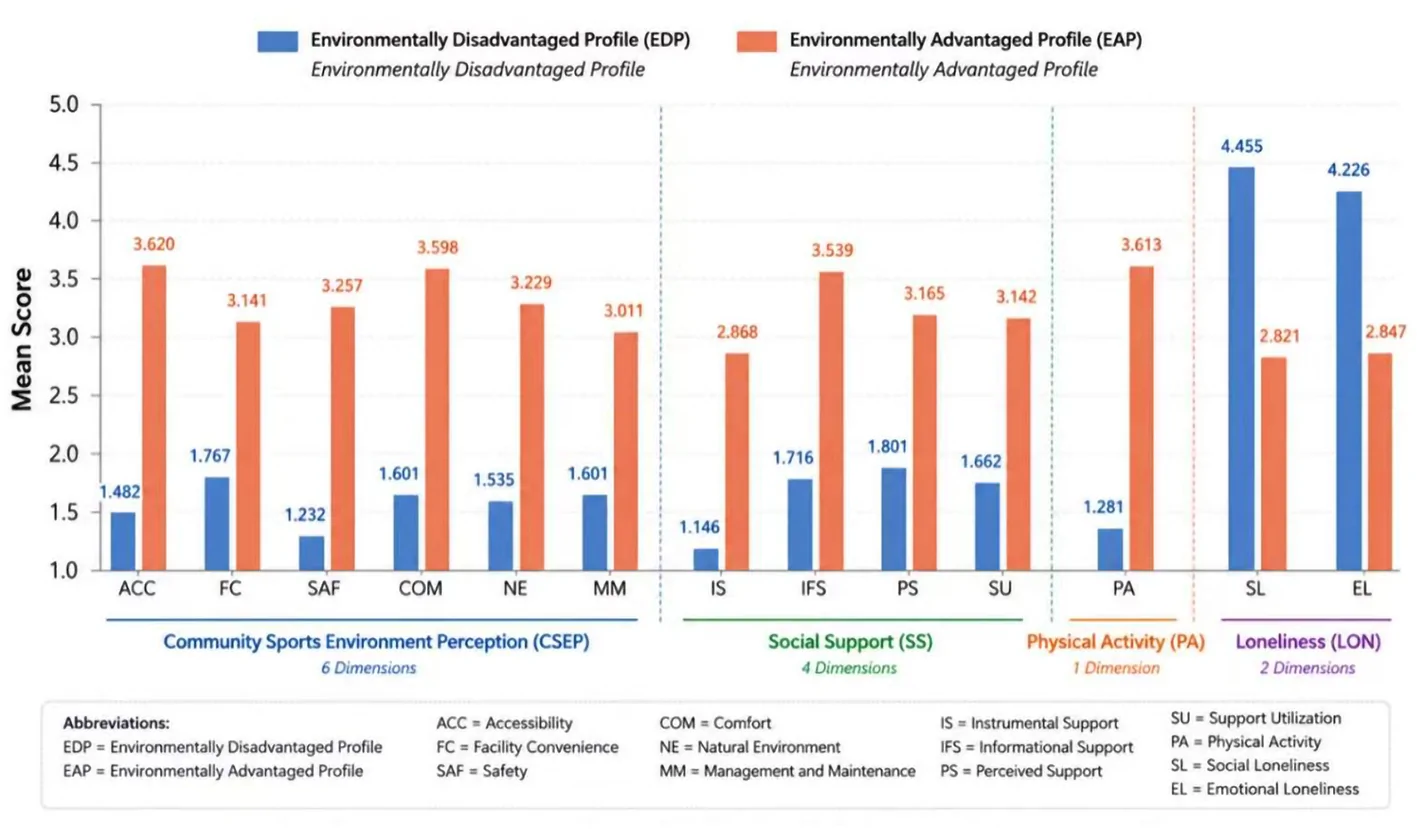
Mean distributions of study variables across different latent profiles.

### 3.6 Network analysis

Figure 3 shows the partial correlation network among the dimensions of perceived community exercise environment, social support, physical activity, and loneliness (58, 59). The network contained 75 nonzero edges, with a network density of 0.9615 (96.15%), indicating a high level of overall connectivity and relatively close conditional associations among nodes. As shown in Figure 3 and Supplementary Table S6, the strongest edge was between social loneliness (SL) and emotional loneliness (EL) (Edge = 0.167), followed by the edge between physical activity (PA) and emotional loneliness (EL) (Edge = −0.149), and the edge between accessibility (ACC) and social loneliness (SL) (Edge = −0.131). Several dimensions of perceived community exercise environment had direct negative connections with loneliness. Specifically, accessibility (ACC) with social loneliness (SL), management and maintenance (MM) with emotional loneliness (EL), facility convenience (FC) with emotional loneliness (EL), comfort (COM) with social loneliness (SL), and safety (SAF) with social loneliness (SL) all showed relatively strong negative associations. These findings suggest that, after controlling for other nodes in the network, more positive perceptions of the community exercise environment were associated with lower levels of social loneliness and emotional loneliness among middle-aged and older adults. Nonparametric bootstrap difference-test results for the network edge weights are presented in Supplementary Figure S1 to show which estimated edge weights differed significantly from one another.

**Figure 3:**
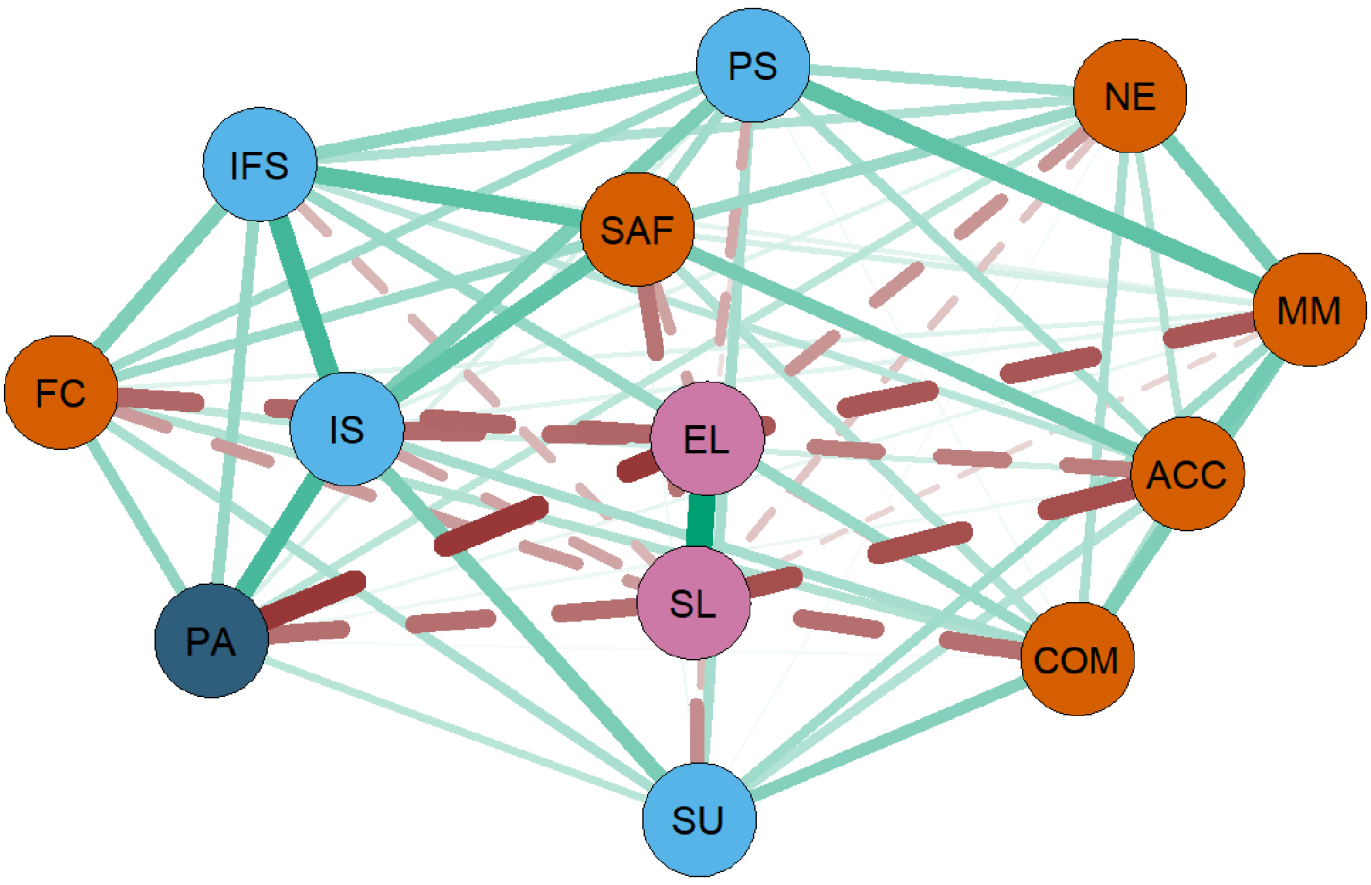
Network structure of perceived community exercise environment, social support, physical activity, and loneliness.

Because the network constructed in this study included both positive and negative associations, expected influence (EI) was used as the node centrality indicator (60). Unlike strength centrality, EI preserves the directional information of edge weights and more accurately reflects the overall influence of each node on the entire network. As shown in Figure 4 and Supplementary Table S7, Figure 4 presents several centrality indices, whereas the narrative focuses on expected influence because it is more appropriate for networks containing both positive and negative edges. Informational support (IFS) had the highest expected influence value (EI = 0.886), indicating that it had the strongest overall positive connections with other nodes in the network. Emotional loneliness (EL) had the lowest expected influence value (EI = −2.269), indicating that it showed the most prominent negative connection pattern and represented the most prominent negative node in the entire network.

**Figure 4:**
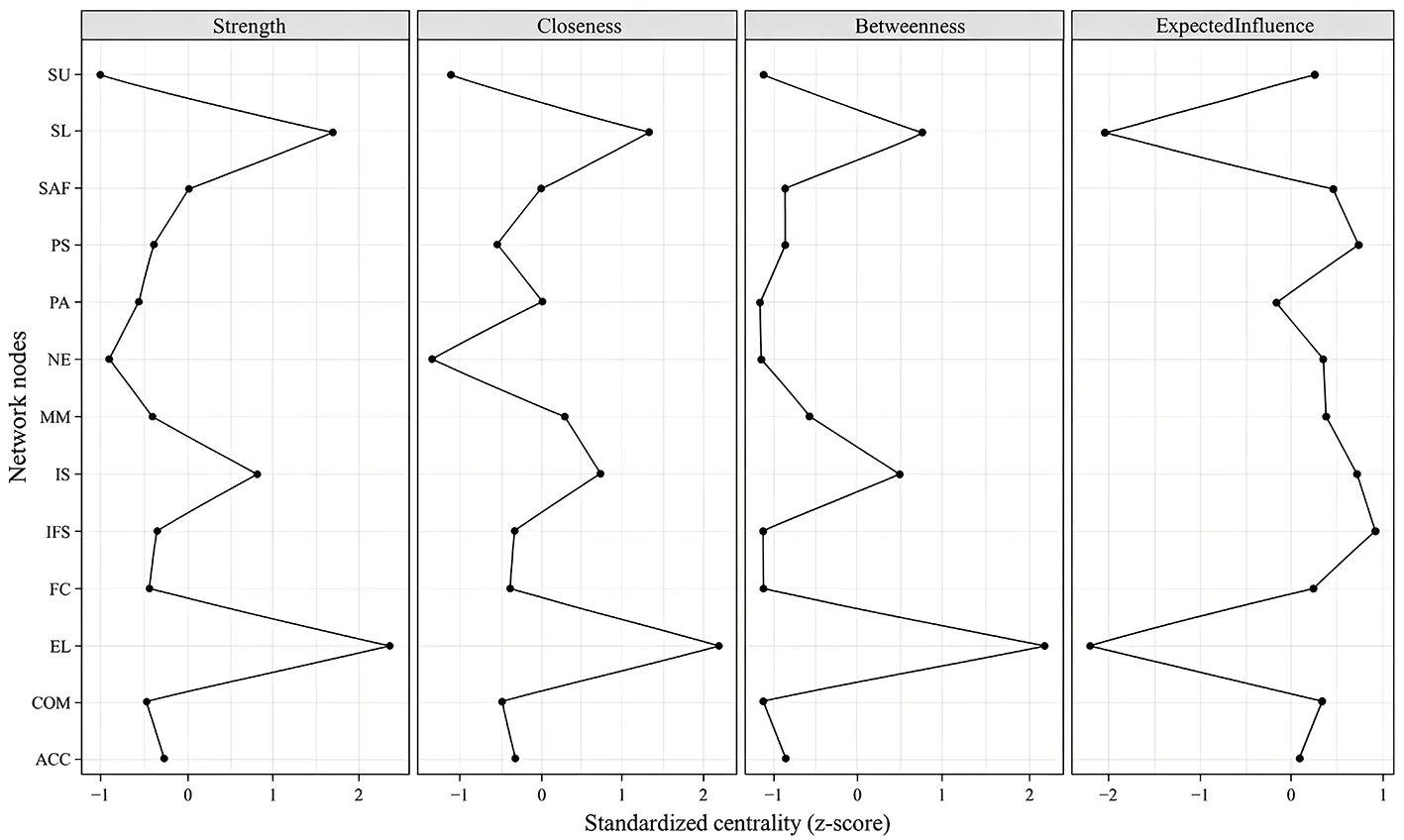
Distribution of network node centrality indicators. ACC, accessibility; FC, facility convenience; SAF, safety; COM, comfort; NE, natural environment; MM, management and maintenance; INS, instrumental support; IFS, informational support; PS, perceived support; SU, support utilization; PA, physical activity; SL, social loneliness; EL, emotional loneliness; EI, expected influence.

To evaluate network robustness, nonparametric bootstrap edge-weight difference testing and case-dropping bootstrap centrality analysis were conducted, with the corresponding results presented in Supplementary Figures S1, S2, respectively. Because expected influence (EI) was used as the node centrality indicator in this study, its stability index was reported as the main result. The correlation stability coefficient (CS-coefficient) for expected influence was 0.439, which was higher than the recommended threshold of 0.25 and close to the ideal threshold of 0.50, indicating good stability of node centrality. In addition, the edge-weight difference test provided complementary information on the statistical distinguishability among the estimated network edges.

## 4 Discussion

### 4.1 Main findings

This study examined the relationship between perceived community exercise environment and loneliness among middle-aged and older adults in Chinese communities. It further tested the roles of social support and physical activity, and used latent profile analysis and network analysis to explore group heterogeneity and dimension-level associations. Taken together, these analyses indicate that loneliness should be understood in relation to the everyday environmental conditions that shape outdoor participation, social contact, and exercise opportunities among middle-aged and older adults. Overall, perceived community exercise environment showed relatively stable associations with social connection, exercise participation, and loneliness experiences among middle-aged and older adults.

These findings suggest that perceived community exercise environment may be linked to loneliness through both direct and indirect pathways. Directly, accessible, safe, comfortable, and well-maintained community exercise spaces may be associated with fewer perceived environmental barriers to going outdoors, more routine participation in public life, and more opportunities for informal neighborhood contact. Indirectly, such environments may be linked to stronger social support because they provide settings for companionship, information exchange, and mutual assistance, and may also be associated with higher physical activity, which is associated with better psychological and social functioning. Because this study used a cross-sectional design, these pathways should be interpreted as statistical associations rather than causal mechanisms.

This study found that more positive perceptions of the community exercise environment were associated with lower levels of loneliness among middle-aged and older adults. This result suggests that loneliness is not only related to insufficient individual social relationships, but may also be shaped by everyday living environments and public activity spaces. In recent years, the concept of 'lonelygenic environments' has been proposed, emphasizing that unfavorable built environments may increase loneliness risk by limiting social contact, weakening belonging, and reducing activity opportunities (61). For middle-aged and older adults, community parks, squares, fitness trails, and open spaces are important places for outdoor activities, physical exercise, and neighborhood interaction. Previous studies have shown that characteristics of neighborhood open spaces are associated with walking and social interaction among older adults (62), and that physical environmental friendliness and access to transportation and services are closely related to social participation among older adults (63). In addition, a review of neighborhood built environments in later life indicated that walkability, service accessibility, community quality, parks, green spaces, and open spaces may influence older adults' health through exercise, socialization, and psychological restoration pathways (64). Therefore, middle-aged and older adults with more positive perceptions of accessible, safe, comfortable, and well-maintained community exercise environments may be more likely to leave home, enter public spaces, and maintain contact with others, which may be one reason for their lower levels of loneliness. This interpretation is consistent with systematic reviews showing that environmental attributes such as safety, walkability, access to destinations and services, recreational facilities, parks, and public open spaces are associated with physical activity and walking among older adults (8, 65). Perceiving community exercise spaces as accessible, safe, and attractive may be associated with fewer barriers to leaving home and more recurring opportunities for outdoor activity, neighborhood contact, and community participation, offering a plausible explanation for the negative association with loneliness observed in this study.

The mediation analysis showed that social support and physical activity had statistical indirect associations in the relationship between perceived community exercise environment and loneliness, including a serial indirect association. This suggests that the community exercise environment may not be associated with loneliness directly only, but may operate through social relational resources and health behavior participation. Community exercise spaces perceived as accessible, safe, comfortable, and well maintained may be associated with more opportunities for middle-aged and older adults to interact with neighbors, peers, and community members, allowing them to obtain instrumental support, informational support, and emotional support through exercising together, exchanging health information, and participating in group activities. A recent systematic review also showed that social networks, social support, social interaction, loneliness, and social environments are all related to physical activity participation among older adults (66). Physical activity itself has been associated with functional ability and social participation among middle-aged and older adults, partly through links with physical function, balance, cardiorespiratory fitness, and overall quality of life (67, 68). Moreover, natural environments and group-based activities may be linked to connectedness, belonging, and social cohesion, making them relevant contextual factors associated with loneliness (69–71). From this perspective, the significance of community exercise spaces lies not only in providing equipment or venues, but also in creating relatively stable settings for daily activity and familiar social interaction among middle-aged and older adults.

This study further used latent profile analysis to examine potential heterogeneity in perceived community exercise environment from a person-centered perspective. The results showed that middle-aged and older adults could be classified into two profiles: the Environmentally Disadvantaged Profile (EDP) and the Environmentally Advantaged Profile (EAP). Compared with traditional variable-centered analysis, latent profile analysis identifies latent groups with similar characteristics based on individuals' score patterns across dimensions, providing a useful perspective for identifying high-risk groups and developing differentiated intervention strategies. The choice of the two-profile model in this study was not based solely on statistical fit indices, but also on classification accuracy, model parsimony, and theoretical interpretability (72, 73). When additional profiles were extracted, they mainly represented further subdivisions of the high environmental perception group and did not form new profiles with clear theoretical differences. Therefore, the two-profile solution more clearly reflected the practical distinction between overall environmental perception advantage and disadvantage. This finding is consistent with previous person-centered research showing that older adults may present distinct social isolation or loneliness-related profiles, highlighting the importance of identifying vulnerable subgroups rather than relying only on average associations.

The profile differences suggest that poorer perceived community exercise environments may represent a broader pattern of environmental and social disadvantage rather than a single facility-related problem. This suggests that perceived community exercise environment not only reflects individuals' evaluations of external exercise environments, but may also capture broader differences in their access to community resources, participation in physical activity, and ability to establish social connections. According to ecological systems theory, community exercise environments characterized by accessibility, convenience, safety, comfort, natural surroundings, and effective management and maintenance may provide residents with safe, convenient, and comfortable activity spaces and may be associated with more opportunities for outdoor activity, neighborhood interaction, social connection, and mental health. In contrast, groups with poorer environmental perceptions may report fewer available exercise resources, lower environmental safety perceptions, and fewer opportunities for community participation, which may be associated with lower physical activity, less social interaction, and higher loneliness. Therefore, for the Environmentally Disadvantaged Profile, future community health promotion and environmental planning may need to consider not only the number of sports facilities, but should also consider whether exercise spaces are easy to reach, safe, well maintained, and supported by activity organizations that can attract sustained participation among middle-aged and older adults.

To further reveal key connections between perceived community exercise environment and loneliness, this study constructed a network analysis model. The results showed that social loneliness and emotional loneliness had the strongest positive association, indicating a high level of co-occurrence between these two loneliness experiences, which is consistent with previous research on the two-dimensional structure of loneliness. More importantly, multiple dimensions of perceived community exercise environment, including accessibility, facility convenience, safety, comfort, and management and maintenance, formed direct negative connections with loneliness. Among them, the associations between accessibility and social loneliness, and between management and maintenance and emotional loneliness, were particularly prominent. These results suggest that the community exercise environment may not only be linked to loneliness indirectly through social support and physical activity, but may also be directly associated with loneliness through daily activity opportunities, community belonging, and neighborhood interaction. This finding complements the serial mediation results and suggests that the association between community exercise environment and loneliness may involve both behavioral and social support pathways, as well as more direct environmental pathways, supporting the ecological perspective on the interaction among environment, behavior, and mental health.

In the network centrality analysis, this study used expected influence as the centrality indicator to preserve information from both positive and negative edge weights (74). The network findings further clarify how specific environmental and psychosocial components are connected within the overall system. The negative connections between accessibility, safety, comfort, management and maintenance, and loneliness suggest that different environmental attributes may correspond to different aspects of loneliness. Accessibility and safety may be more closely related to social loneliness because they shape whether middle-aged and older adults can regularly enter public spaces and maintain neighborhood contact. In contrast, management and maintenance and facility convenience may be more closely related to emotional loneliness because well-maintained and usable exercise spaces may be associated with a stronger sense of security, comfort, and belonging in everyday community life. This distinction suggests two related but different pathways: one pathway links environmental conditions to opportunities for social participation, and the other links environmental experience to subjective emotional connectedness. Emotional loneliness, as the most prominent negative node, may reflect the subjective affective dimension of disconnection, whereas social loneliness may be more closely tied to participation opportunities and the availability of social ties. Therefore, the network results should not be interpreted only as a ranking of nodes, but as evidence that environmental perceptions and different loneliness dimensions are embedded in a broader system of social and behavioral associations. This interpretation is consistent with ecological perspectives on aging and health, which emphasize that physical environments, social participation, and psychological wellbeing are interrelated in later life.

Overall, the three types of analysis produced generally consistent findings: more positive perceptions of community exercise environments, including accessibility, facility convenience, safety, comfort, natural surroundings, and management and maintenance, were usually accompanied by higher social support, higher physical activity, and lower loneliness. The serial mediation analysis provided evidence of indirect statistical associations among variables, latent profile analysis identified the Environmentally Disadvantaged Profile as a group requiring particular attention, and network analysis further suggested that accessibility, management and maintenance, informational support, and emotional loneliness may have relatively high relevance for future research and community health promotion. Together, these results indicate that optimizing community exercise environments should be combined with physical activity promotion and social support building, rather than being limited to the construction of exercise facilities alone; These findings also highlight the relevance of planning, maintenance, and activation of public exercise spaces for shared activity and everyday social interaction.

### 4.2 Practical implications for active aging and community exercise environments

This study has several practical implications for the development of community exercise environments in the context of active aging. First, community exercise spaces may be understood not merely as sports facilities, but as community resources associated with physical activity, social participation, and psychological wellbeing among middle-aged and older adults. Related evidence from social prescribing research suggests that linking individuals to community activities and community activity resources has been associated with social participation and physical and mental health among older adults, although higher-quality evidence is still needed (75). Second, the optimization of community exercise environments should move beyond the question of whether facilities are available and pay greater attention to whether these spaces are accessible, safe, comfortable, and sustainable for daily use. This emphasis is also consistent with the environmental domain of the WHOQOL-BREF, which considers safety, accessibility to services and opportunities, and participation in meaningful activities as important components of environmental quality of life (76). For example, better walking paths and barrier-free facilities, adequate lighting and anti-slip conditions, and stronger facility maintenance and order management may be associated with more favorable environmental experiences of middle-aged and older adults and reduce barriers to outdoor activity. Third, community exercise programs should be integrated with social support building. Low-threshold group activities, such as walking groups, tai chi, square dancing, fitness exercises, and shuttlecock kicking, may provide opportunities for peer companionship, volunteer guidance, health information exchange, and regular neighborhood interaction. In this sense, community exercise spaces should not only support physical activity participation, but also serve as everyday settings related to social connection and lower loneliness among middle-aged and older adults.

### 4.3 Limitations and future research

This study has several limitations. First, this study used a cross-sectional questionnaire survey design. Although the serial mediation and network analyses revealed statistical associations among variables, they could not determine temporal order or causal direction. Future studies could use longitudinal follow-up, natural experiments, or community intervention designs to examine temporal relationships between community exercise environments, loneliness, social support, and physical activity participation. Second, this study mainly used self-report scales, which may be affected by recall bias and social desirability bias. Third, although the survey covered six cities in different regions of China and the recruitment procedures were generally consistent across sites, convenience sampling was used and the sample size varied slightly across cities. Potential regional differences in community exercise environments, public space use, and loneliness should therefore be considered when interpreting the findings. Fourth, this study focused mainly on community exercise environments, social support, physical activity, and loneliness, but did not collect detailed information on other daily activities, such as employment status, work-related demands, caregiving responsibilities, household activities, or participation in non-exercise community activities. These factors may influence both exposure to community exercise spaces and experiences of loneliness, especially among middle-aged adults who may still be engaged in paid work or family caregiving. Future studies should include broader daily activity indicators to better understand how community exercise environments are embedded in the everyday lives of middle-aged and older adults. Future research could combine objective built environment indicators, geographic information system (GIS) data, such as spatial measures of park accessibility, walking-route distance, service facilities, and neighborhood land-use characteristics, wearable device records, and community activity participation records to improve the objectivity of the results. Future research could also use qualitative interviews, mixed-methods designs, or larger quantitative surveys to identify which specific components of community exercise environments are perceived as disadvantages by middle-aged and older adults. Such research would help clarify whether accessibility, facility convenience, safety, comfort, natural environment, or management and maintenance are the most salient barriers in different community contexts, and could provide more targeted evidence for future community planning and health-promotion efforts. Fifth, the participants in this study were mainly middle-aged and older adults with experience in community exercise participation. The applicability of the findings to older adults who are long-term homebound, mobility-limited, living in institutional care, or residing in rural areas requires further verification. Sixth, the latent profile and network results may be influenced by sample characteristics, indicator selection, and model specification. Future studies should examine the stability of the classification and the replicability of the network in larger samples and across different types of communities. In particular, network analysis should pay attention to issues of replicability and dynamics, and further use longitudinal networks or ecological momentary assessment methods to reveal temporal dynamics among community environment, social support, physical activity, and loneliness.

## 5 Conclusion

Based on a sample of middle-aged and older adults in Chinese communities, this study examined the relationship between perceived community exercise environment and loneliness, and further analyzed this relationship from three perspectives: statistical indirect associations, group heterogeneity, and network structure. These findings suggest that the community exercise environment is not only a physical space for physical activity among middle-aged and older adults, but also an important ecological condition for supporting social interaction, social support, and lower loneliness. Overall, the study indicates that perceived community exercise environments are linked to loneliness through the combined roles of environmental quality, social connection, and physical activity, highlighting their relevance as community-level resources for active aging.

## Data Availability

The original contributions presented in the study are included in the article/Supplementary material, further inquiries can be directed to the corresponding author.
