# Supplementary material for: Perceived community exercise environment and loneliness among middle-aged and older adults in China: psychosocial-behavioral associations, latent profiles, and network analysis
Source: Front Public Health. 2026 Sep 21;14:1925992. doi: 10.3389/fpubh.2026.1925992 (PMC13634969; doi:10.3389/fpubh.2026.1925992)
Supplement: Supplementary file 1 [file Supplementary_File_1.docx]

Supplementary Material

# 1 Supplementary Figures and Tables

## **1.1 Scale development and validation of the perceived community exercise environment scale**

Table S1. Items and scoring instructions for the Perceived Community Exercise Environment Scale.

Scoring instructions: Each item is rated on a 5-point Likert scale from 1 = strongly disagree to 5 = strongly agree. All items are positively scored. Higher scores indicate more positive perceptions of the community exercise environment. The total score is calculated as the mean of all 18 items. Dimension scores are calculated as the mean of the 3 items within each dimension.

| **Item** | **Dimension** | **Item content** |
| --- | --- | --- |
| 1 | Accessibility | It is convenient for me to travel from home to the community exercise place I usually use. |
| 2 | Accessibility | The location of community exercise places makes it convenient for me to exercise regularly. |
| 3 | Accessibility | I can easily reach parks, squares, or fitness trails suitable for exercise. |
| 4 | Facility convenience | There are places in the community suitable for older adults to exercise. |
| 5 | Facility convenience | The fitness equipment in the community can meet my exercise needs. |
| 6 | Facility convenience | There are roads or paths in the community suitable for walking, jogging, or other activities. |
| 7 | Safety | I feel safe when exercising in the community. |
| 8 | Safety | The ground surface of community exercise places is relatively even and not easy to fall on. |
| 9 | Safety | The lighting conditions of community exercise places can meet the needs of morning or evening exercise. |
| 10 | Comfort | The community exercise environment is generally clean and tidy. |
| 11 | Comfort | I do not feel that the community exercise space is too crowded when exercising. |
| 12 | Comfort | The community exercise environment has relatively low noise and is suitable for exercise and relaxation. |
| 13 | Natural environment | The community greenery makes me feel relaxed. |
| 14 | Natural environment | The air, trees, or landscape in the community make me feel pleasant. |
| 15 | Natural environment | Community exercise places have good shade, sun protection, or natural landscape features. |
| 16 | Management and maintenance | The community exercise equipment is well maintained. |
| 17 | Management and maintenance | The order of community exercise places is good. |
| 18 | Management and maintenance | The community can provide good public services for residents’ exercise. |

Item analysis: Critical-ratio (CR) analysis and item-total correlation analysis were performed for the 18-item perceived community exercise environment scale. Items with |CR| < 3.0 or item-total correlation < 0.40 would be removed. All items met the retention criteria. The results are presented in Table S2.

Table S2. Item analysis results for the perceived community exercise environment scale.

| **Dimension** | **Item** | **CR (t)** | **Item-total correlation (r)** |
| --- | --- | --- | --- |
| Accessibility | CSEP1 | -11.029*** | 0.560*** |
| Accessibility | CSEP2 | -9.549*** | 0.545*** |
| Accessibility | CSEP3 | -11.432*** | 0.587*** |
| Facility convenience | CSEP4 | -8.887*** | 0.489*** |
| Facility convenience | CSEP5 | -9.261*** | 0.511*** |
| Facility convenience | CSEP6 | -9.284*** | 0.518*** |
| Safety | CSEP7 | -10.020*** | 0.512*** |
| Safety | CSEP8 | -10.693*** | 0.539*** |
| Safety | CSEP9 | -10.254*** | 0.528*** |
| Comfort | CSEP10 | -11.374*** | 0.513*** |
| Comfort | CSEP11 | -10.636*** | 0.528*** |
| Comfort | CSEP12 | -10.427*** | 0.518*** |
| Natural environment | CSEP13 | -7.438*** | 0.472*** |
| Natural environment | CSEP14 | -7.638*** | 0.511*** |
| Natural environment | CSEP15 | -6.876*** | 0.475*** |
| Management and maintenance | CSEP16 | -8.682*** | 0.503*** |
| Management and maintenance | CSEP17 | -10.298*** | 0.523*** |
| Management and maintenance | CSEP18 | -9.317*** | 0.510*** |

Note: ***p < 0.001. CSEP = perceived community exercise environment item.

Internal consistency: Reliability analysis was conducted for the total scale and the six dimensions. The pretest and formal-sample results are shown in Table S3.

**Table S3. Reliability analysis of the perceived community exercise environment scale.**

| **Dimension** | **Number of items** | **Cronbach's alpha (pretest)** | **Cronbach's alpha (formal sample)** |
| --- | --- | --- | --- |
| Accessibility | 3 | 0.930 | 0.878 |
| Facility convenience | 3 | 0.917 | 0.867 |
| Safety | 3 | 0.916 | 0.875 |
| Comfort | 3 | 0.928 | 0.861 |
| Natural environment | 3 | 0.915 | 0.912 |
| Management and maintenance | 3 | 0.939 | 0.902 |
| Overall scale | 18 | 0.841 | 0.852 |

Exploratory structure analysis: Principal component analysis with varimax rotation was conducted as an initial structural exploration. The KMO value was 0.757, and Bartlett's test of sphericity was significant (p < 0.001), indicating that the data were suitable for factor extraction. Six components with eigenvalues greater than 1 were extracted, and the cumulative variance explained was 87.18%.

**Table S4. KMO and Bartlett's test.**

| **Test** | **Value** |
| --- | --- |
| Kaiser-Meyer-Olkin measure of sampling adequacy | 0.757 |
| Bartlett's test of sphericity: Approx. Chi-square | 3535.921 |
| df | 153 |
| p | <0.001 |

**Table S5. Rotated component matrix for the perceived community exercise environment scale.**

| **Item** | **Factor 1** | **Factor 2** | **Factor 3** | **Factor 4** | **Factor 5** | **Factor 6** |
| --- | --- | --- | --- | --- | --- | --- |
| CSEP1 |  | 0.911 |  |  |  |  |
| CSEP2 |  | 0.924 |  |  |  |  |
| CSEP3 |  | 0.908 |  |  |  |  |
| CSEP4 |  |  |  | 0.916 |  |  |
| CSEP5 |  |  |  | 0.906 |  |  |
| CSEP6 |  |  |  | 0.913 |  |  |
| CSEP7 |  |  |  |  |  | 0.911 |
| CSEP8 |  |  |  |  |  | 0.909 |
| CSEP9 |  |  |  |  |  | 0.895 |
| CSEP10 |  |  | 0.927 |  |  |  |
| CSEP11 |  |  | 0.926 |  |  |  |
| CSEP12 |  |  | 0.912 |  |  |  |
| CSEP13 |  |  |  |  | 0.925 |  |
| CSEP14 |  |  |  |  | 0.902 |  |
| CSEP15 |  |  |  |  | 0.908 |  |
| CSEP16 | 0.938 |  |  |  |  |  |
| CSEP17 | 0.929 |  |  |  |  |  |
| CSEP18 | 0.930 |  |  |  |  |  |

Note: Extraction method = principal component analysis; rotation method = varimax with Kaiser normalization. Loadings below 0.40 are not displayed.

Confirmatory factor analysis: A six-factor model was constructed for the perceived community exercise environment scale. The model fit indices (χ²/df = 1.893, RMSEA = 0.061, SRMR = 0.053, CFI = 0.969, TLI = 0.961) indicated acceptable structural validity. The CFA model is shown in Figure S3.

## **1.2 Network analysis supplementary results**

Only nodes from the present study were retained in the revised network tables: ACC, FC, SAF, COM, NE, MM, IS, IFS, PS, SU, PA, SL, and EL. Rows containing variables from unrelated projects were removed.

Table S6. Major edge weights in the cross-sectional network. Nonparametric bootstrap difference-test results for the network edge weights are shown in Figure S1.

| **Rank** | **Node 1** | **Node 2** | **Edge weight** |
| --- | --- | --- | --- |
| 1 | SL | EL | 0.167 |
| 2 | PA | EL | -0.149 |
| 3 | ACC | SL | -0.131 |
| 4 | MM | EL | -0.126 |
| 5 | IS | IFS | 0.125 |
| 6 | IS | PA | 0.120 |
| 7 | FC | EL | -0.114 |
| 8 | IS | EL | -0.112 |
| 9 | SAF | IFS | 0.108 |
| 10 | COM | SL | -0.107 |
| 11 | PA | SL | -0.107 |
| 12 | MM | PS | 0.105 |
| 13 | SAF | IS | 0.104 |
| 14 | SAF | SL | -0.103 |
| 15 | ACC | EL | -0.096 |
| 16 | ACC | MM | 0.092 |
| 17 | ACC | SAF | 0.089 |
| 18 | NE | MM | 0.089 |
| 19 | IS | SU | 0.087 |
| 20 | IS | PS | 0.087 |
| 21 | COM | MM | 0.085 |
| 22 | SU | SL | -0.085 |
| 23 | FC | IFS | 0.085 |

Note: ACC = Accessibility; FC = Facility Convenience; SAF = Safety; COM = Comfort; NE = Natural Environment; MM = Management and Maintenance; IS = Instrumental Support; IFS = Informational Support; PS = Perceived Support; SU = Support Utilization; PA = Physical Activity; SL = Social Loneliness; EL = Emotional Loneliness.

**Table S7. Expected influence scores for each node in the network.**

| **Rank** | **Node** | **Expected influence** |
| --- | --- | --- |
| 1 | IFS | 0.886 |
| 2 | PS | 0.717 |
| 3 | IS | 0.715 |
| 4 | SAF | 0.471 |
| 5 | MM | 0.388 |
| 6 | NE | 0.364 |
| 7 | COM | 0.341 |
| 8 | SU | 0.258 |
| 9 | FC | 0.231 |
| 10 | ACC | 0.099 |
| 11 | PA | -0.134 |
| 12 | SL | -2.067 |
| 13 | EL | -2.269 |

## **1.3 Supplementary figures**

Figure S1. Nonparametric bootstrap difference test for network edge weights. The matrix presents pairwise bootstrap difference tests among the estimated edge weights.

Figure S2. Case-dropping bootstrap stability results for node centrality indices.

Figure S3. Six-factor confirmatory factor analysis model of the perceived community exercise environment scale.

Table S8. Sensitivity analysis using the conventional PARS-3 product score.

| **Analysis** | **Variables** | **Statistic** | **Result** |
| --- | --- | --- | --- |
| Score correspondence | PARS-3 mean score and PARS-3 product score | Pearson r | 0.960, p < 0.001 |
| Correlation pattern | Perceived community exercise environment and PARS-3 product score | Pearson r | 0.247, p < 0.001 |
| Correlation pattern | Social support and PARS-3 product score | Pearson r | 0.274, p < 0.001 |
| Correlation pattern | PARS-3 product score and loneliness | Pearson r | -0.332, p < 0.001 |
| Interpretation | Comparison with main scoring approach | Conclusion | The direction of associations was consistent with the main analysis using the mean PARS-3 score. |

Note: The conventional PARS-3 product score was calculated as exercise intensity × exercise duration × exercise frequency. The sensitivity analysis was conducted to evaluate whether the modified mean-score approach changed the substantive pattern of associations.

**Supplementary Note. Development of the Perceived Community Exercise Environment Scale**

The perceived community exercise environment scale was developed through five steps: literature review, qualitative interviews, Delphi expert consultation, pilot testing, and final item revision. First, based on the social ecological model, age-friendly environment framework, built environment research, community sports studies, and previous studies on physical activity among older adults, the research team identified six preliminary dimensions of perceived community exercise environment: accessibility, facility convenience, safety, comfort, natural environment, and management and maintenance. An initial item pool was then generated according to these dimensions.

Second, semi-structured qualitative interviews were conducted with 25 community-dwelling middle-aged and older adults who had experience participating in community-based exercise. The interviewees were recruited from community parks, squares, fitness trails, outdoor fitness equipment areas, and other public exercise spaces. The interviews focused on participants’ daily exercise settings, convenience of access, facility use experience, perceived safety, environmental comfort, natural landscape perception, site order, equipment maintenance, and public service provision. Based on the interview data, the research team revised the initial item pool by deleting redundant items, simplifying unclear wording, and modifying items that did not adequately reflect the actual exercise experiences of middle-aged and older adults.

Third, two rounds of Delphi expert consultation were conducted to evaluate the dimensions and items. In the first round, 20 experts participated. The experts had backgrounds in sports science, ageing and public health, community sports, exercise psychology, sociology, urban community environment, public space research, scale development, or statistical measurement. The experts evaluated each item in terms of theoretical relevance, content representativeness, clarity of wording, dimensional appropriateness, practical applicability, and comprehensibility for middle-aged and older adults. Based on the first-round expert feedback, several items were merged, deleted, reworded, or reassigned to more appropriate dimensions.

Fourth, 15 experts participated in the second round of Delphi consultation. The second round focused on the revised items and further assessed their importance, clarity, representativeness, and dimensional appropriateness. Items were retained, revised, or deleted according to the following criteria: theoretical relevance to perceived community exercise environment, representativeness of the intended dimension, clarity and simplicity of wording, consistency of expert opinions, avoidance of item redundancy, suitability for middle-aged and older adults, and feedback from the pilot test.

Finally, a pilot test was conducted before the formal survey to examine item comprehensibility, response time, internal consistency, and preliminary measurement performance. The pilot sample was independent of the final analytical sample. Based on pilot-test feedback and preliminary statistical results, minor wording revisions were made. The final scale included 18 items across six dimensions. All items were rated on a 5-point Likert scale, with higher scores indicating more positive perceptions of the community exercise environment.

**Supplementary Table S9. Basic information of Delphi experts involved in scale development.**

| **Expert category** | **First round** | **Second round** | **Main expertise** |
| --- | --- | --- | --- |
| Sports science experts | 5 | 4 | Sports sociology, community sports, public sports services |
| Ageing and public health experts | 4 | 3 | Healthy ageing, public health, health behavior |
| Community sports practitioners | 3 | 2 | Community sports services, grassroots sports organization |
| Exercise psychology or social psychology experts | 3 | 2 | Social support, loneliness, exercise psychology |
| Urban community environment or public space experts | 3 | 2 | Built environment, public space, age-friendly community environment |
| Scale development and statistical measurement experts | 2 | 2 | Questionnaire development, psychometrics, statistical analysis |
| Total | 20 | 15 | — |

**Supplementary Table S10. Criteria for item retention, revision, or deletion during scale development.**

| **Criterion** | **Description** | **Item decision** |
| --- | --- | --- |
| Theoretical relevance | Whether the item reflected the theoretical meaning of perceived community exercise environment | Items with weak relevance were revised or deleted |
| Content representativeness | Whether the item represented the intended dimension | Items with insufficient representativeness were revised or reassigned |
| Clarity of wording | Whether the item was clear, concise, and easy for middle-aged and older adults to understand | Ambiguous items were reworded |
| Dimensional appropriateness | Whether the item matched the assigned dimension | Items with unclear dimensional attribution were modified or reassigned |
| Expert agreement | Whether experts showed sufficient agreement on the importance and appropriateness of the item | Items with low agreement were discussed, revised, or deleted |
| Redundancy | Whether the item overlapped substantially with other items | Redundant items were merged or removed |
| Pilot-test feedback | Whether participants understood the item and completed it smoothly | Items with poor feedback were revised or removed |
| Preliminary item performance | Whether the item showed acceptable discrimination and contribution to internal consistency | Items with weak performance were further reviewed |
